# Two New Species of *Betacixius* Matsumura, 1914 (Hemiptera: Fulgoromorpha: Cixiidae) from Southwestern China, with an Updated Checklist and Key to Species †

**DOI:** 10.3390/insects13060512

**Published:** 2022-05-30

**Authors:** Yan Zhi, Xiao-Ya Wang, Lin Yang, Xiang-Sheng Chen

**Affiliations:** 1 Laboratory Animal Center, Guizhou Medical University, Guiyang 550025, China; zhiyan0428@163.com; 2 Institute of Entomology, Guizhou University, Guiyang 550025, China; wangxy541@163.com (X.-Y.W.); yanglin6626@163.com (L.Y.); 3 The Provincial Special Key Laboratory for Development and Utilization of Insect Resources of Guizhou, Guizhou University, Guiyang 550025, China

**Keywords:** Auchenorrhyncha, new species, planthopper, taxonomy

## Abstract

**Simple Summary:**

*Betacixius* Matsumura, 1914 is a small genus of cixiid planthoppers distributed throughout China, Japan and Vietnam. Despite its rich biodiversity in Southwest China, *Betacixius* has not been taxonomically well studied in this region. Here, two new species, *Betacixius* *gongshanensis* sp. nov. from Yunan Province and *B. guizhouensis* sp. nov. from Guizhou Province, are described, giving the genus 27 species in total. We believe that the discovery in this study will contribute to further studies on the classification and phylogeny of Cixiidae.

**Abstract:**

In this study, two new species of genus *Betacixius* Matsumura, 1914 (Fulgoromorpha, Cixiidae), *Betacixius gongshanensis* sp. nov. from Yunnan Province and *B. guizhouensis* sp. nov. from Guizhou Province, are described and illustrated. An updated checklist and identification key to known species of the genus *Betacixius* are provided.

## 1. Introduction

The cixiid planthopper genus *Betacixius* Matsumura, 1914, in the tribe Semonini (Hemiptera: Cixiidae: Cixiinae), currently consists of 25 species and two subspecies distributed throughout China, Japan and Vietnam [1]. Following our previous works [2,3], we aim to revise the species from Southwest China in the present study. The specimens from Guizhou and Yunnan provinces brought to our attention another two new species, *Betacixius gongshanensis* and *B. guizhouensis,* which are described and illustrated here. The total number of *Betacixius* species is thus increased to 27, with 25 occurring in China. An updated checklist and identification key of *Betacixius* are given.

## 2. Materials and Methods

The morphological terminology follows Bourgoin [4] for male genitalia, Bourgoin et al. [5] for wing venation, and Bourgoin [6] for female genitalia. Body length was measured from the apex of the vertex to the tip of the forewing; vertex length represented the median length of the vertex (from the apical transverse carina to the tip of basal emargination). Fuchsin staining was used to highlight the female genitalia structures we studied. External morphology and drawings were visualized and created with the aid of a Leica MZ 12.5 stereomicroscope. Photographs were taken with the KEYENCE VHX-6000 system. Illustrations were scanned with a CanoScan LiDE 200 and imported into Adobe Photoshop C7.0 for labeling and plate composition. The dissected male and female genitalia are preserved in glycerin in small plastic tubes pinned together with the specimens. Zoogeographic regionalization scheme follows Holt et al. [7]. The distribution map was prepared with Simplemappr (http://www.simplemappr.net accessed on 7 March 2022).

The type specimens were deposited in the Institute of Entomology, Guizhou University, Guiyang, Guizhou Province, China (GUGC).

Institutional abbreviations

CAS = California Academy of Sciences, San Francisco, USA.

EUM = Entomological Laboratory, College of Agriculture, Ehime University, Matsuyama, Japan.

GUGC = Institute of Entomology, Guizhou University, Guiyang, Guizhou, China.

HU = Hokkaido University, Sapporo, Japan.

MTD = State Museum of Zoology, Dresden, Germany.

NCHU = National Chung Hsing University, Taiwan, China.

NTU = National Taiwan University, Taiwan, China.

TARI = Taiwan Agricultural Research Institute, Taiwan, China.

ZFMK = Museum Alexander Koenig, Bonn, Germany.

## 3. Results

### 3.1. Taxonomy

*Betacixius* Matsumura, 1914

*Betacixius* Matsumura 1914: 412; Tsaur et al. 1991: 27; Zhang and Chen 2011; Zhi et al. 2020.

Type species: *Betacixius ocellatus* Matsumura, 1914, by original designation.

For diagnosis of *Betacixius*, see Zhang and Chen [2].

Distribution. China, Japan, Vietnam.

Key to species of *Betacixius* Matsumura, 1914

1 Forewing with markings·············································································································2
  - Forewing without any markings ··························································································23
2 Forewing with a large ocellate marking in apical half···························································3
  - Forewing without ocellate marking in apical half ······························································6
3 Forewing with an oblique, brown band extending from the clavus across the middle of corium························································································*B. tonkinensis* Matsumura, 1914
  - Forewing without such a band····························································································4
4 Endosoma (=flagellum) of aedeagus with one spine, hook-shaped···················································································*B. flagellihamus* Zhang & Chen, 2011
  - Endosoma of aedeagus with two spines, not hook-shaped··············································5
5 Periandrium of aedeagus apically with two L-shaped processes···················································································*B. maculosus* Tsaur & Hsu, 1991
  - Periandrium of aedeagus apically with one nearly straight and one arched processes·······················································································*B. ocellatus* Matsumura, 1914
6 Forewing with an oblique band extending from stigma passing through its middle part ··························································································································································7
  - Forewing without such a band·································································································13
7 Forewing with apical cells of M and Cu strongly infuscate *B. transversus* Jacobi, 1944
  - Forewing with apical cells not infuscate················································································8
8 Forewing with apical margin black or distinctly darkened······················································9
  - Forewing with apical margin fuscous or not distinctly darkened······································10
9 Frons with a pallid spot at centre of lateral margins; mesonotum testaceous·····················································································*B. kumejimae* Matsumura, 1914
  - Frons without such spots; mesonotum, except scutellum, castaneous-piceous···································································································*B. euterpe* Fennah, 1956
10 Forewing with a spot near sutural margin of clavus near union of claval veins, no oblique dark band at this level extending into corium···········································································11
  - Forewing with an oblique dark band extending from clavus into centre of corium, slightly distad of level of union of claval veins······································································12
11 Forewing basally with a broad transverse band from dorsal margin to sutural margin of clavus··························································································*B. latissimus* Zhi & Chen, 2020
  - Forewing without above band ···············································*B. obliquus* Matsumura, 1914
12 Forewing basally with a light brown band ·······································*B. pallidior* Jacobi, 1944
  - Forewing basally without band ·····························································*B. michioi* Hori, 1982
13 Forewing with a long black stripe from base, along clavus extending to Rs·········································································································*B. fuscus* Tsaur & Hsu, 1991
  - Forewing without such a stripe ····························································································14
14 Forewing along the R with a black stripe widened towards the rear ····················································································································*B. robustus* Jacobi, 1944
  - Forewing without such a stripe ·······················································································15
15 Anal segment asymmetrical·································································*B. nelides* Fennah, 1956
  - Anal segment symmetrical··································································································16
16 Ventral margin of periandrium basally with two broad, lobate processes··················································································*B. bispinus* Zhang & Chen, 2011
  - Ventral margin of periandrium basally without process ·················································17
17 Endosoma apically without spinose process·········································································18
  - Endosoma apically with one or two spinose processes·························································19
18 Spinose process on right side of periandrium medium-sized, curved upwards, apex dorsally directed; spinose process on left side parallel to periandrium for most potion, apex ventrocephalically directed·······················································*B. rinkihonis* Matsumura, 1914
  - Spinose process on right side of periandrium very short, nearly straight, apex directed cephalad; spinose process on left side generally dorsocephalically directed······································································································*B. shirozui* Hori, 1982
19 In lateral view, apical lobe of anal segment ventrally rounded··············································20
  - In lateral view, apical lobe of anal segment ventrally pointed············································21
20 Both spinose processes of periandrium curved downwards ·················································································································*B. gongshanensis* sp. nov.
  - Both spinose processes of periandrium curved upwards······················································································*B. delicatus* Tsaur & Hsu, 1991
21 Spinose process on left side of periandrium curved from left to right side over periandrium and apex exceeded right lateral margin of periandrium ················································································································ *B. guizhouensis* sp. nov.
  - Apex of spinose process on left side of periandrium not exceeded right lateral margin of periandrium ··························································································································22
22 Spinose process on right side of periandrium near dorsal margin, coiled 90 degrees to left; endosoma with two spinose processes··········································*B. sparsus* Tsaur & Hsu, 1991
  - Spinose process on right side of periandrium near ventral margin, nearly straight, apex directed cephalad; endosoma with one spinose process ································································································*B. maguanensis* Zhi & Chen, 2020
23 Endosoma of aedeagus apically with two processes ································································24
  - Endosoma of aedeagus apically with one process ··························································· 25
24 Ventral margin of periandrium with a long process ······················ *B. flavovittatus* Hori, 1982
  - Ventral margin of periandrium without process············ *B. nigromarginalis* Fennah, 1956
25 Frons without median carina·························································· *B. clypealis* Matsumura, 1914
  - Frons with median carina ····································································································26
26 Body pale brown; periandrium of aedeagus with two processes on right side ······································································································ *B. brunneus* Matsumura, 1914
  - Body green; periandrium of aedeagus with one process on right side····························································································*B. herbaceus* Tsaur & Hsu, 1991

#### 3.1.1. *Betacixius gongshanensis* Zhi & Chen sp. nov.

##### urn:lsid:zoobank.org:act:6C2FAED6-17D6-479B-A26B-88432284CF0C

**Figure 1:**
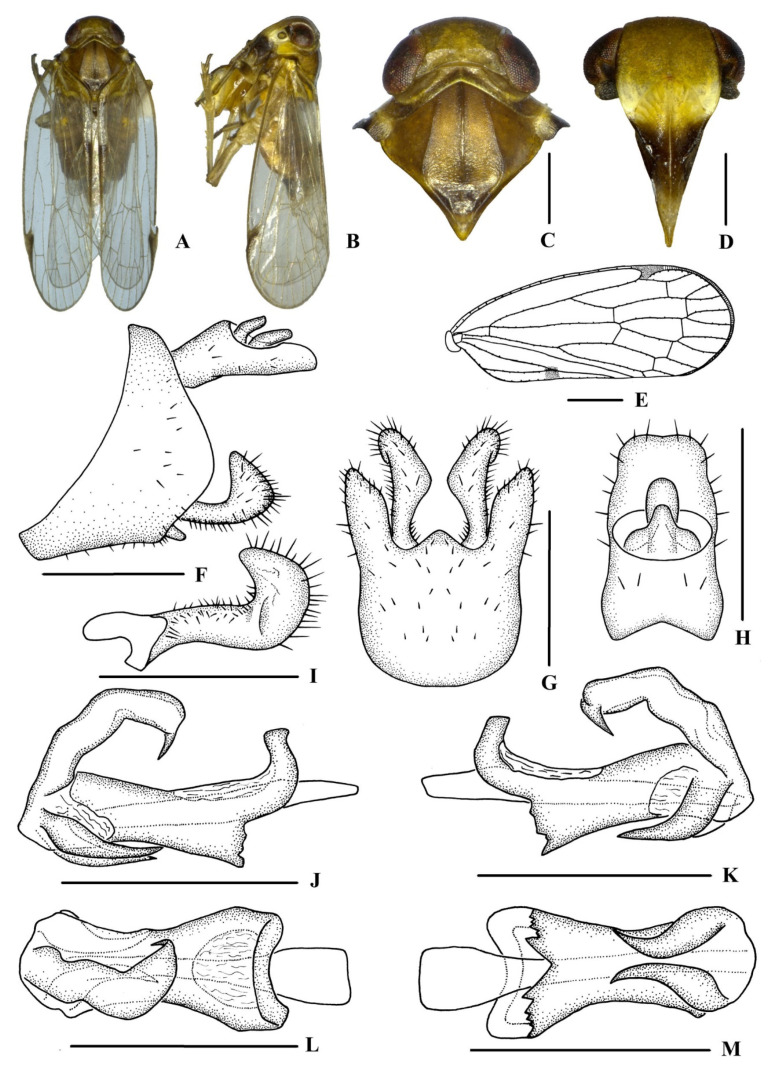
*Betacixius gongshanensis* Zhi & Chen, sp. nov., male (**A**,**B**) habitus, dorsal (**A**) and lateral (**B**) views; (**C**) head and thorax, dorsal view; (**D**) face, ventral view; (**E**) forewing; (**F**) genitalia, lateral view; (**G**) pygofer and gonostyli, ventral view; (**H**) anal segment, dorsal view; (**I**) gonostyli, inner lateral view; (**J**–**M**) aedeagus, right lateral (**J**), left lateral (**K**), dorsal(**L**) and ventral (**M**) views. Scale bars: 0.5 mm (**C**,**D**,**F**–**M**); 1.0 mm (**E**).

**Figure 2:**
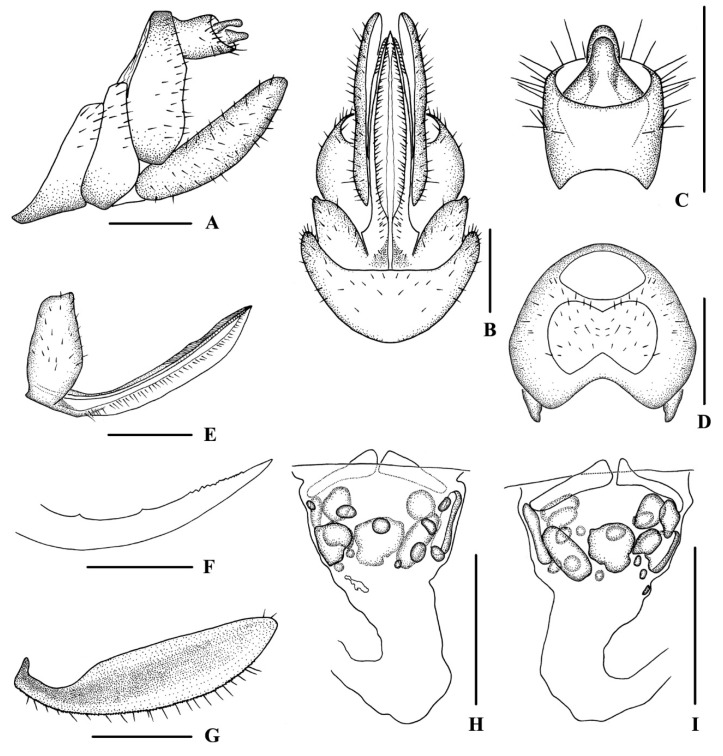
*Betacixius gongshanensis* Zhi & Chen, sp. nov., female. (**A**,**B**) genitalia, lateral (**A**) and ventral (**B**) views; (**C**) anal segment, dorsal view; (**D**) tergite IX, caudal view; (**E**) gonapophysis VIII and gonocoxa VIII, ventral view; (**F**) gonapophysis IX, lateral view; (**G**) gonoplac, inner lateral view; (**H**,**I**) posterior vagina, ventral (**H**) and dorsal (**I**) views. Scale bar: 0.5 mm.

Figure 1 and Figure 2

**Type Material. Holotype.** ♂ China, Yunnan Province, Gongshan County, Bingzhongluo Town, 28°1′ N, 98°39′ E, 9 May 2010, Pei Zhang, Jun-Qiang Ni, Yan-Li Zheng, Hu Li, Bin Zhang leg. (GUGC). **Paratypes.** 8 ♂♂, 7 ♀♀, same data as for holotype (GUGC).

**Description.** Measurements. Body length: male 5.1–5.7 mm (*N* = 9), female 5.7–6.7 mm (*N* = 7).

*Coloration*. General color yellowish brown (Figure 1A–D). Eyes dark brown, ocelli dark red. Vertex yellowish to dark brown, pronotum and mesonotum brown. Frons generally yellowish brown, with a whitish yellow marking at areas of level below median ocellus, above frontoclypeal suture, extending to antennae, lateral part of pronotum and base of forewing. Postclypeus yellow to blackish brown and anteclypeus blackish brown. Rostrum generally yellowish brown. Forewing semi-translucent, clavus with a blackish brown spot on apical third, stigma blackish brown. Hind tibiae yellowish brown and abdominal sternites blackish brown.

*Head and thorax*. Vertex (Figure 1A,C) broad, 1.7 times wider than long; anterior margin arched convexly, posterior margin arched concavely; median carina distinct and complete. Frons (Figure 1D) 0.6 times as long as wide, median carina indistinct, extending from slightly above level of lateral ocelli to median ocellus. Clypeus with median carina distinct and elevated throughout. Pronotum (Figure 1C) 1.1 times longer than vertex, posterior margin concave at an obtuse angle. Mesonotum 1.6 times longer than pronotum and vertex combined. Forewing (Figure 1E) 2.6 times longer than wide, with nine apical and five subapical cells; fork Sc+RP slightly distad of fork CuA_1_+CuA_2_; first crossvein r-m slightly basad of fork MP; RP two branches, MP with four terminals: MP_1_, MP_2_, MP_3_, and MP_4_, fork MP_1_+MP_2_ distad of fork MP_3_+MP_4_. Hind tibia with three lateral spines, metatibiotarsal formula: 6/7–8/7–8, second segment of the hind tarsus with four platellae.

*Male genitalia*. Pygofer (Figure 1F,G) symmetrical, dorsal margin concave and U-shaped ventrally, widening towards apex; in lateral view, lateral lobes arched and extended caudally. Medioventral process triangular in ventral view. Anal segment (Figure 1F,H) tubular and symmetrical, with apical lobes ventrally round in lateral view, 1.9 times longer than wide in dorsal view; anal style strap-like, not beyond anal segment. Gonostyli (Figure 1F,G,I) symmetrical in ventral view, in inner lateral view, apical part extended in a triangle. Aedeagus (Figure 1J–M) with three processes. Left and right sides of the periandrium apically with a medium-sized spinose process, both spinose processes curved downwards, with the middle part closer to each other under the periandrium and the apexes directed outwards respectively. Endosoma (=flagellum) slender, structure simple, apex with a small hook-like spinose process, ventrocaudally directed.

*Female genitalia*. Tergite IX (Figure 2A,B,D) moderately sclerotized, with a large wax plate, nearly oval, dorsal and ventral margins concave. Anal segment (Figure 2A,C) rectangular, 1.2 times wider than long in dorsal view, anal style strap-like. Gonapophysis VIII (Figure 2E) elongated, and slightly curved upwards. Gonapophysis IX (Figure 2F) with two middle teeth, with a distance ratio, between distal middle tooth to apex and length of denticulate portion, of 2.4. Gonoplac (Figure 2G) rod-like, 3.9 times longer than wide in lateral view. Posterior vagina pattern, as shown in Figure 2H,I is elongated. All sclerites located on the basal half of posterior vagina. Ventral wall with several round and oval sclerites dispersed, with a large one on the left side and the others being relatively small; dorsal wall with oval and round sclerites that larger than the ones on ventral wall.

**Etymology.** The species name is derived from Gongshan County, Yunan Province, where the type locality is located.

**Distribution.** China: Yunnan.

**Remarks.***Betacixius gongshanensis* Zhi & Chen sp. nov. is similar to *B. maguanensis* Zhi & Chen, 2020; however, it differs in that: (1) the left spinose process of the periandrium curves downwards (the same spinose process is directed upwards in *B. maguanensis*); (2) the apical lobes of the anal segment are round in the lateral view (in *B. maguanensis*, the apical lobes of the anal segment are pointed in the lateral view); and (3) the mesonotum is brown (while the latter is black).

**Diagnosis.** This species can be distinguished from other species of the same genus by the following combination of characteristics: the pronotum and mesonotum are both brown; the clavus of the forewing has a blackish brown spot on the apical third; the anal segment is symmetrical, with the apical lobes ventrally round in the lateral view; the aedeagus with the apexes of the left and right sides of the periandrium, each with a medium-sized spinose process, curves downwards, with the middle parts closer to each other under the periandrium and the apexes directed outwards, respectively; and finally the apex of the endosoma has a small hook-like spinose process.

#### 3.1.2. *Betacixius guizhouensis* Zhi & Chen sp. nov.

##### urn:lsid:zoobank.org:act:43F7FB24-67D3-4AD3-AD4E-A15DA77C053E

**Figure 3:**
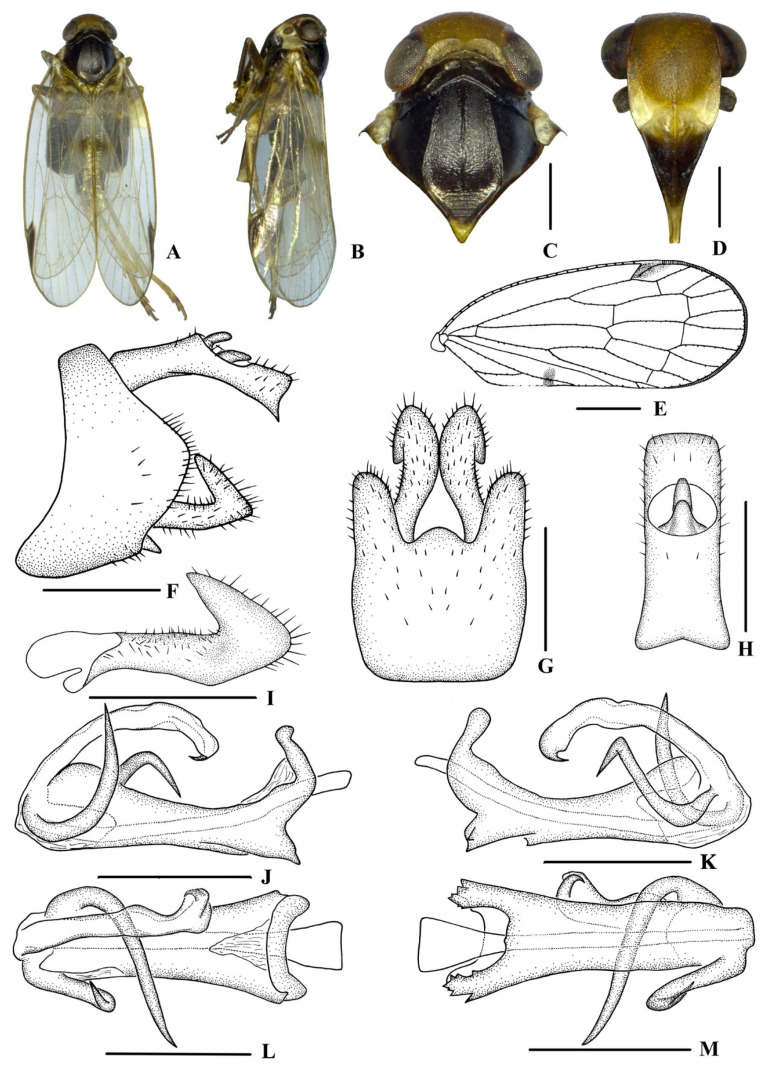
*Betacixius guizhouensis* Zhi & Chen, sp. nov., male (**A, B**) habitus, dorsal (**A**) and lateral (**B**) views; (**C**) head and thorax, dorsal view; (**D**) face, ventral view; (**E**) forewing; (**F**) genitalia, lateral view; (**G**) pygofer and gonostyli, ventral view; (**H**) anal segment, dorsal view; (**I**) gonostyli, inner lateral view; (**J**–**M**) aedeagus, right lateral (**J**), left lateral (**K**), dorsal(**L**) and ventral (**M**) views. Scale bars: 0.5 mm (**C**–**D**,**F**–**M**); 1.0 mm (**E**).

**Figure 4:**
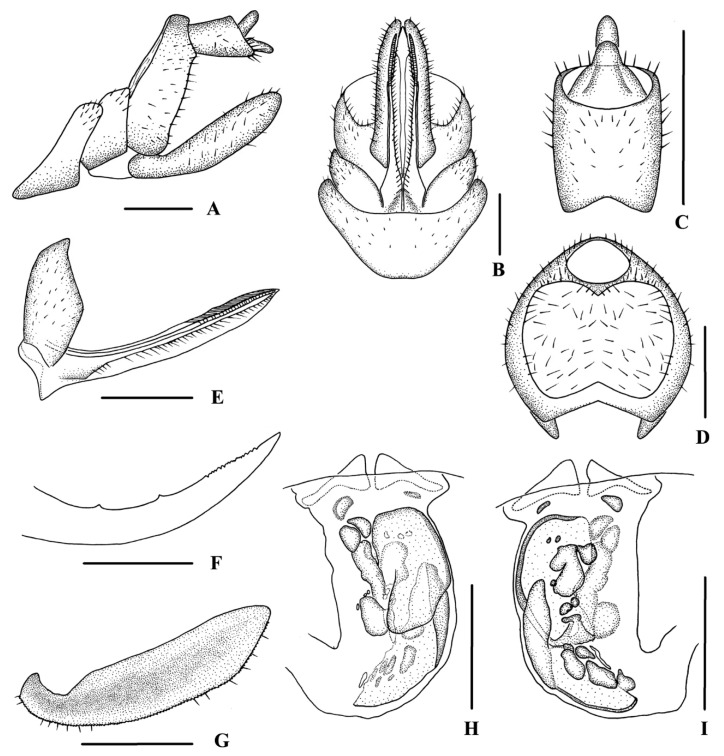
*Betacixius guizhouensis* Zhi & Chen, sp. nov., female. (**A**,**B**) genitalia, lateral (**A**) and ventral (**B**) views; (**C**) anal segment, dorsal view; (**D**) tergite IX, caudal view; (**E**) gonapophysis VIII and gonocoxa VIII, ventral view; (**F**) gonapophysis IX, lateral view; (**G**) gonoplac, inner lateral view; (**H**,**I**) posterior vagina, ventral (**H**) and dorsal (**I**) views. Scale bars: 0.5 mm.

Figure 3 and Figure 4

**Type Material. Holotype.** ♂ China, Guizhou Province, Daozhen County, Xiannvdong Nature Reserve, alt. 600–700 m, 29°3′ N, 107°25′ E, 25–27 May 2004, Xiang-Sheng Chen leg. (GUGC). **Paratypes.** 1 ♂, 5 ♀♀, same data as for holotype (GUGC), 1 ♂; Guizhou Province, Libo County, Maolan Town, Sanchahe, 25°19′ N, 108°0′ E, 9 April 2004, Pei Zhang, Jian-Kun Long leg. (GUGC).

**Description.** Measurements. Body length: male 5.1–6.3 mm (*N* = 3), female 5.7–7.4 mm (*N* = 5).

*Coloration*. General color blackish brown (Figure 3A–D). Eyes dark brown, ocelli dark red. Vertex yellowish brown, pronotum and mesonotum black. Frons generally yellowish brown, with a whitish yellow marking below the median ocellus, above frontoclypeal suture extending to antennae, lateral parts of pronotum and base of the forewing. Postclypeus yellow to blackish brown and anteclypeus blackish brown. Rostrum generally brown. Forewing semi-translucent, clavus with a blackish brown spot on apical third, stigma blackish brown. Hind tibiae brown and abdominal sternites blackish brown.

*Head and thorax*. Vertex (Figure 3A,C) broad, 1.9 times wider than long; anterior margin arched convexly, posterior margin arched concavely, median carina distinct and complete. Frons (Figure 3D) with a length nearly equal to its width, median carina indistinct, extending from slightly above level of lateral ocelli towards median ocellus. Clypeus with median carina distinct and elevated throughout. Pronotum (Figure 3C) 1.5 times longer than vertex, and the posterior margin concave at an obtuse angle. Mesonotum 1.5 times longer than pronotum and vertex combined. Forewing (Figure 3E) 2.5 times longer than wide, with nine apical and five subapical cells; fork Sc+RP slightly distad of fork CuA_1_+CuA_2_; first crossvein r-m slightly distad of fork MP; RP two branches, MP with four terminals, MP_1_, MP_2_, MP_3_, and MP_4_, fork MP_1_+MP_2_ distad of fork MP_3_+MP_4_. Hind tibia with three lateral spines, metatibiotarsal formula: 6/7/7, second segment of hind tarsus with three platellae.

*Male genitalia*. Pygofer (Figure 3F,G) symmetrical, with dorsal margin concave and U-shaped ventrally and widened towards apex; in lateral view, lateral lobes arch extend caudally. Medioventral process arc in ventral view. Anal segment (Figure 3F,H) long, tubular, and symmetrical, with apical lobes ventrally pointed in lateral view, and 2.3 times longer than wide in dorsal view; anal style finger-like, and not beyond the anal segment. Gonostyli (Figure 3F,G, I) symmetrical in ventral view; in inner lateral view, apical part extended and triangular. Aedeagus (Figure 3J–M) with three processes. Right side of periandrium with a long spinose process at the apex, which strongly curved upwards, and the apex dorsally directed; spinose process on left side of periandrium being the longest, gently curving from left to right over periandrium, with apex exceeding right lateral margin of periandrium and being right-ventrocephalically directed. Endosoma (=flagellum) slender, structure simple, and the apex with a small hook-like spinose process.

*Female genitalia*. Tergite IX (Figure 4A,B,D) moderately sclerotized, with a large oval wax plate, dorsal and ventral margins concave. Anal segment (Figure 4C) rectangular, 1.4 times wider than long in dorsal view, with anal style finger-like. Gonapophysis VIII (Figure 4E) elongated, and slightly curved upwards. Gonapophysis IX (Figure 4F) with two middle teeth, at a distance ratio, between distal middle tooth to apex and length of denticulate portion, of 2.3. Gonoplac (Figure 4G) rod-like, 3.7 times longer than wide in lateral view. Posterior vagina pattern as shown in Figure 4H,I, elongated. Ventral wall of posterior vagina with two large oval sclerites on the right: the basal one with the left 1/4 cracked and the other one with the right basal 1/2 bent towards the dorsal wall. Dorsal wall with several small, round, oval, and irregular sclerites arranged longitudinally in the middle area.

**Etymology.** The species name is derived from Guizhou Province, where the type locality is located.

**Distribution.** China: Guizhou.

**Remarks.** The male genitalia of *Betacixius guizhouensis* Zhi & Chen sp. nov. is similar to that of *B. rinkihonis* Matsumura, 1914, but differs in: (1) the apical lobes of the anal segment are pointed in the lateral view (in *B. rinkihonis*, the apical lobes of the anal segment are round in the lateral view); (2) the endosoma has a small hook-like spinose process apically (the latter does not have this spinose process); and (3) the anal segment is 2.3 times longer than it is wide (the anal segment is only 1.5 times longer than it is wide in *B. rinkihonis*).

**Diagnosis.** This species can be distinguished from other species of the genus by the following combination of characteristics: the pronotum and mesonotum are black; the clavus of the forewing has a blackish brown spot on the apical third; the anal segment is symmetrical, with the apical lobes ventrally pointed in the lateral view; the apexes of the left and right sides of the periandrium each have a long spinose process, with the right spinose process being strongly curved upwards, and the left one being straighter and gently curving from left to right over the periandrium, and the apex exceeding the right lateral margin of the periandrium; and the apex of endosoma has a small hook-like spinose process.

#### 3.1.3. Checklist and Distributions of the Species of *Betacixius* Matsumura, 1914

*B. bispinus* Zhang & Chen, 2011

*Betacixius bispinus* Zhang & Chen, 2011: 53; Holotype: ♂ (GUGC); type locality: China (Guizhou: Yanhe County).

Distribution. China: Guangxi, Guizhou, Sichuan, Xinjiang, Yunnan.

*B. brunneus* Matsumura, 1914

*Betacixius brunneus* Matsumura, 1914: 417; Lectotype: ♂ (HU), designated by Liang and Suwa [8]; type locality: China (Taiwan: Jiayi County).

Distribution. China: Fujian, Taiwan, Zhejiang; Japan: Ryukyu Islands.

*B. clypealis* Matsumura, 1914

*Betacixius clypealis* Matsumura, 1914: 415; Lectotype: ♀ (HU), designated by Liang and Suwa [8]; type locality: China (Taiwan: Jiayi County).

Distribution. China: Zhejiang, Taiwan.

*B. clypealis vitifrons* (Matsumura, 1914)

*Betacixius clypealis vitifrons* (Matsumura, 1914): 416; Lectotype: ♂ (HU), designated by Liang and Suwa [8]; type locality: China (Taiwan: Jiayi County).

Distribution. China: Taiwan.

*B. delicatus* Tsaur & Hsu, 1991

*Betacixius delicatus* Tsaur & Hsu in Tsaur et al., 1991: 29; Holotype: ♂ (NCHU); type locality: China (Taiwan: Pingdong County).

Distribution. China: Shaanxi, Taiwan., Yunnan, Zhejiang,

*B. euterpe* Fennah, 1956

*Betacixius euterpe* Fennah, 1956: 458, Holotype: ♂ (CAS); type locality: China (Guangdong: Lechang City).

Distribution. China: Guangdong.

*B. flagellihamu*s Zhang & Chen, 2011

*Betacixius flagellihamu*s Zhang & Chen, 2011: 54; Holotype: ♂ (GUGC); type locality: China (Guizhou: Leishan County).

Distribution. China: Guizhou.

*B. flavovittatus* Hori, 1982

*Betacixius flavovittatus* Hori, 1982: 179. Holotype: ♂ (EUM); type locality: China (Taiwan: Nantou County).

Distribution. China: Zhejiang, Taiwan.

*B. fuscus* Tsaur & Hsu, 1991

*Betacixius fuscus* Tsaur & Hsu in Tsaur et al., 1991: 44. Holotype: ♂ (TARI); type locality: China (Taiwan: Hualian County).

Distribution. China: Fujian, Taiwan.

*B. gongshanensis* Zhi & Chen sp. nov.

Holotype: ♂ (GUGC); type locality: China (Yunnan: Gongshan County).

**Figure 5:**
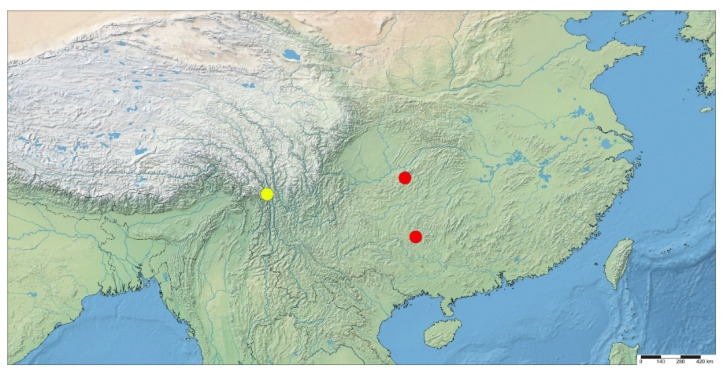
Distribution records of *Betacixius gongshanensis* sp. nov. (yellow circle) and *B. guizhouensis* sp. nov. (red circle).

Distribution. China: Yunnan (Figure 5).

*B. guizhouensis* Zhi & Chen sp. nov.

Holotype: ♂ (GUGC); type locality: China (Guizhou: Daozhen County).

Distribution. China: Guizhou (Figure 5).

*B. herbaceus* Tsaur & Hsu, 1991

*Betacixius herbaceus* Tsaur & Hsu in Tsaur et al., 1991: 28; Holotype: ♂ (NTU); type locality: China (Taiwan: Yilan County).

Distribution. China: Yunnan, Taiwan.

*B. kumejimae* Matsumura, 1914

*Betacixius kumejimae* Matsumura, 1914: 415; Lectotype: ♀ (HU), designated by Liang and Suwa [8]; type locality: Japan (Okinawa).

Distribution. Japan: Ryukyu Islands.

*B. latissimus* Zhi & Chen, 2020

*Betacixius latissimus* Zhi & Chen in Zhi et al., 2020: 8; Holotype: ♂ (GUGC); type locality: China (Yunnan: Jinping County).

Distribution. China: Yunnan.

*B. maculosus* Tsaur & Hsu, 1991

*Betacixius maculosus* Tsaur & Hsu in Tsaur et al., 1991: 31; Holotype: ♂ (NCHU); type locality: China (Taiwan: Hualian County).

Distribution. China: Fujian, Sichuan, Taiwan.

*B. maguanensis* Zhi & Chen, 2020

*Betacixius maguanensis* Zhi & Chen in Zhi et al., 2020: 11; Holotype: ♂ (GUGC); type locality: China (Yunnan: Maguan County).

Distribution. China: Yunnan.

*B. michioi* Hori, 1982

*Betacixius michioi* Hori, 1982: 176; Holotype: ♂ (EUM); type locality: China (Taiwan: Nantou County).

Distribution. China: Yunnan, Taiwan.

*B. nelides atrior* Fennah, 1956

*Betacixius nelides atrior* Fennah, 1956: 458, Holotype: ♂ (CAS); type locality: China (Zhejiang: Hangzhou City).

Distribution. China: Zhejiang.

*B. nelides nelides* Fennah, 1956

*Betacixius nelides nelides* Fennah, 1956: 457; Holotype: ♂ (CAS); type locality: China (Zhejiang: Tonglu County).

Distribution. China: Zhejiang.

*B. nigromarginalis* Fennah, 1956

*Betacixius nigromarginalis* Fennah, 1956: 457; Holotype: ♂ (CAS); type locality: China (Hubei: Lichuan City).

Distribution. China: Hubei.

*B. obliquus* Matsumura, 1914 

*Betacixius obliquus* Matsumura, 1914: 414; Lectotype: ♀ (HU), designated by Liang and Suwa [8]; type locality: Japan (Honshu).

Distribution. China: Fujian, Guizhou, Guangxi, Guangdong, Hainan, Hunan, Sichuan, Yunnan, Zhejiang; Japan: Honshu, Kyushu, Shikoku.

*B. ocellatus* Matsumura, 1914

*Betacixius ocellatus* Matsumura, 1914: 412; Lectotype: ♀ (HU), designated by Tsaur et al. [9]; type locality: China (Taiwan).

Distribution. China: Fujian, Taiwan, Yunnan.

*B. pallidior* Jacobi, 1944

*Betacixius pallidior* Jacobi, 1944: 15; Syntype: 10♂♀ (ZFMK, MTD); type locality: China (Fujian: Shaowu City).

Distribution. China: Fujian; Vietnam: Hanoi.

*B. rinkihonis* Matsumura, 1914

*Betacixius rinkihonis* Matsumura, 1914: 417; Lectotype: ♂ (HU), designated by Tsaur et al. [9]; type locality: China (Taiwan).

Distribution. China: Guangdong, Taiwan.

*B. robustus* Jacobi, 1944

*Betacixius robustus* Jacobi, 1944: 15; Syntype: 6♂♀ (ZFMK, MTD); type locality: China (Fujian: Guadun).

Distribution. China: Fujian.

*B. shirozui* Hori, 1982

*Betacixius shirozui* Hori, 1982: 178. Holotype: ♂ (EUM); type locality: China (Taiwan: Jiayi County).

Distribution. China: Yunnan, Taiwan.

*B. sparsus* Tsaur & Hsu, 1991

*Betacixius sparsus* Tsaur & Hsu in Tsaur et al., 1991: 46; Holotype: ♂ (NTU); type locality: China (Taiwan: Taidong County).

Distribution. China: Fujian, Guangxi, Hainan, Taiwan.

*B. tonkinensis* Matsumura, 1914

*Betacixius tonkinensis* Matsumura, 1914: 413; Lectotype: ♂ (HU), designated by Liang and Suwa [8]; type locality: Vietnam: (Tonkin, Montes Mauson).

Distribution. Vietnam: Lang Son.

*B. transversus* Jacobi, 1944

*Betacixius transversus* Jacobi, 1944: 14; Syntype: ♂♀ (ZFMK, MTD); type locality: China (Fujian: Guadun).

Distribution. China: Fujian.

Remarks. Distribution data were collected from Matsumura [10], Jacobi [11], Fennah [12], Tsaur et al. [9], Hori [13] Liang and Suwa [8], Zhang and Chen [2], Hayashi and Fujinuma [14], Zhi et al. [3] and Luo et al. [15].

## 4. Discussion

The genus *Betacixius* Matsumura, 1914, belongs to the tribe Semonini (Hemiptera: Cixiidae: Cixiinae), which is characterized by a swollen postclypeus, a convex clypeofrontal suture, and incomplete or obscure median carina of frons [16,17]. Morphologically, *Betacixius* may be easily distinguished from other genera of Semonini by the presence of 4–5 subapical cells, 8–9 apical cells on the forewing, a vertex much wider than it is long at the midline, and a chaetotaxy of the hind tarsus 7–8/7–8.

The vagina in Cixiidae was described in general by Bourgoin [6]. The sclerites situated on the walls of the posterior vagina are considered to have high diagnostic value at the species level [16,18,19,20]. We found that the characteristics of the posterior vaginal walls can provide evidence for the species diagnosis of *Betacixius* species in both this study and that of Zhi et al. [3]. The morphological characteristics of the posterior vagina should be given more attention through detailed descriptions and illustrations in future research.

Based on data from published information as well as from the present study, *Betacixius* presents a distribution pattern in the Sino-Japanese and Oriental biogeographic regions. The discovery of two new species in Southwest China suggests that the current species richness of the genus remains underestimated. Further collecting and investigation of *Betacixius* taxa are doubtless required to understand its real diversity.

## Data Availability

All data are available in this paper.
